# Sleep quality and sleep duration predict brain microstructure among community-dwelling older adults

**DOI:** 10.1016/j.neurobiolaging.2023.02.001

**Published:** 2023-02-10

**Authors:** Amaryllis A. Tsiknia, Humberto Parada, Sarah J. Banks, Emilie T. Reas

**Affiliations:** a Department of Neurosciences, University of California, San Diego, La Jolla, CA, USA; b Division of Epidemiology and Biostatistics, San Diego State University, San Diego, CA, USA

**Keywords:** Sleep quality, Sleep duration, Brain microstructure, Aging, Diffusion MRI

## Abstract

Although poor sleep quality and extreme sleep durations have been associated with brain atrophy and dementia, it is unclear whether sleep disturbances contribute to neural injury in the absence of neurodegeneration and cognitive impairment. In 146 dementia-free older adults of the Rancho Bernardo Study of Healthy Aging (76.7 ± 7.8 years at MRI), we examined associations of restriction spectrum imaging metrics of brain microstructure with self-reported sleep quality 6.3 ± 0.7 years prior, and with sleep duration reported 25, 15 and 9 years prior. Worse sleep quality predicted lower white matter restricted isotropic diffusion and neurite density and higher amygdala free water, with stronger associations between poor sleep quality and abnormal microstructure for men. Among women only, short or long sleep duration 25 and 15 years before MRI predicted lower white matter restricted isotropic diffusion and increased free water. Associations persisted after accounting for associated health and lifestyle factors. Sleep patterns were not related to brain volume or cortical thickness. Optimizing sleep behaviors throughout the life-course may help to preserve healthy brain aging.

## 1. Introduction

Cognitive aging trajectories are modified by health and lifestyle, with an estimated 40% of risk for dementia potentially explained by modifiable factors (Livingston et al., 2020). While there is strong consensus that certain behaviors and characteristics, such as physical activity or education, are beneficial for preserving brain health, the role of sleep remains unclear. Despite evidence linking sleep disturbances with higher rates of dementia (Shi et al., 2018) and accumulation of Alzheimer’s disease (AD) neuropathology, specific sleep patterns that may be deleterious for brain function, and optimal sleep recommendations have not been established due to inconsistent findings and prevailing uncertainties (Livingston et al., 2020).

Sleep regulates crucial restorative functions that maintain homeostasis of a healthy neural environment, including glymphatic clearance of waste products, cellular repair, and controlling inflammation (Benveniste et al., 2019; Hafycz and Naidoo, 2019). Impairment of these processes by disrupted sleep may contribute to the neurodegenerative changes that underlie cognitive decline with age. Sleep disruptions may shorten or lengthen nightly sleep duration, both of which have been linked to higher rates of dementia. Prior studies have implicated a potential “sweet-spot” of sleep duration, reporting lowest risk estimates for 7–8 hours of sleep nightly (Sabia et al., 2021; Yaffe et al., 2014). However, brain changes associated with normal and pathological aging may also hinder sleep quality (Hafycz and Naidoo, 2019; Ju et al., 2014). Considering the hypothesized bidirectional relationship between sleep disturbances and brain aging (Lo et al., 2014; Ramduny et al., 2022; Sabia et al., 2021; Sexton et al., 2014), efforts to identify effects of sleep behaviors on brain outcomes should focus on cognitively intact populations with sufficient lag between sleep assessment and subsequent imaging to minimize confounding by latent pathology.

While several studies have found associations of poor sleep quality and short sleep duration with widespread cortical atrophy (Lo et al., 2014; Sexton et al., 2014; Spira et al., 2016) and white matter (WM) lesions (Ramos et al., 2014), effects of sleep on microstructural brain changes that precede detectable neurodegeneration, are not well characterized. Among the few diffusion MRI studies that have probed this question, 2 found associations of poor sleep quality with microstructural abnormalities over a 5-year period (Kocevska et al., 2019) and with increased hippocampal diffusivity that was driven by amyloid burden and mediated memory decline (Grydeland et al., 2021), whereas another with a longer follow-up reported no association (Sexton et al., 2017). Notably, the former 2 studies assessed sleep quality with the well-validated Pittsburg Sleep Quality Index (PSQI) questionnaire (Buysse et al., 1989), while the latter used the 4-item Jenkins Sleep Scale (Jenkins et al., 1988), which may provide a less precise measure of sleep quality.

Despite some evidence for sex differences in associations between sleep apnea or sleep loss and WM integrity (Macey et al., 2012) or cognition (Hajali et al., 2019), sex differences in associations of sleep duration and quality with brain aging have not been sufficiently examined. Further research into the modifying effects of sex will be instrumental in guiding personalized approaches to minimize the adverse effects of sleep disruption on brain aging. Furthermore, sleep patterns are tightly linked to biological and lifestyle factors that evolve over the lifespan and influence brain aging trajectories. Determining how sleep shapes brain health will demand careful consideration of confounding and modifying factors.

To identify microstructural brain properties in later life associated with prior sleep patterns beginning in midlife, we used restriction spectrum imaging (RSI), a multicompartment diffusion MRI technique which dissociates diffusion fractions from intracellular, intraneurite, extracellular, and free water tissue compartments. We previously reported sensitivity of RSI to cytoarchitectural abnormalities that not only predict AD pathology and future cognitive decline in early AD (Reas et al., 2018, 2017), but also correlate with chronological age (Reas et al., 2020), cognition (Reas et al., 2021a), and vascular dysfunction (Reas et al., 2021b) in cognitively normal older adults. Herein, we apply RSI to determine if prior sleep behaviors predict brain microstructure among a well-characterized cohort of older adults without dementia from the Rancho Bernardo Study of Healthy Aging (RBS). First, we examined whether prior sleep quality predicted brain microstructure an average of 6 years later and identified the most predictive sleep quality components. We next examined relationships between sleep duration at multiple earlier life periods and brain microstructure. Sleep duration was assessed up to 30 years prior to RSI, presenting a unique opportunity to evaluate prospective associations between sleep duration from midlife to late-life, and brain cytoarchitecture in older age. Finally, exploratory analyses assessed potential sex-differences in associations between prior sleep patterns and brain microstructure. We expected worse sleep quality and extreme sleep durations to be associated with microstructural brain abnormalities, indexed by higher free water and lower intracellular diffusion.

## 2. Methods and methods

### 2.1. Participants

Participants were members of the RBS, which included 12 research assessments from study inception in 1972–1974 to the final visit in 2014–2016. Participants who completed an MRI in 2014–2016 (visit 12) and either the PSQI in 2008 or self-reported sleep duration at least once and up to 6 times from 1984 to 2009 (visits 4–11) were eligible for study inclusion (*N* = 154). Exclusion criteria included history of head injury, stroke, neurological disease, treatment for an alcohol use disorder, or safety contraindication for MRI. After excluding 1 participant due to missing sleep data, 6 due to poor imaging data quality, and 1 due to severe WM disease, the final sample included 146 participants (62% women; age at MRI: mean ± SD 76.7 ± 7.8, range 56–99 years) with available MRI data and either a PSQI score or at least 1 measure of self-reported sleep duration. Study procedures were approved by the University of California, San Diego Human Research Protections Program Board and participants provided informed written consent. The RBS data are publicly available at https://knit.ucsd.edu/ranchobernardostudy/. Raw image data are available from the investigators upon request.

### 2.2. Sleep assessment

Sleep quality was assessed in 2008 using the PSQI (Buysse et al., 1989), a 19-item questionnaire assessing general sleep habits over the past month. Responses are combined to generate 7 component scores (0–3 points), including subjective sleep quality, sleep latency, sleep duration, habitual sleep efficiency, sleep disturbances, use of sleeping medication, and daytime dysfunction. The total PSQI score is calculated as the sum of these component scores (0–21 points), with higher scores indicating worse sleep quality.

Over a maximum of 6 research visits in 1984–1987 (visit 4), 1992–1996 (visit 7), 1997–1999 (visit 8), 1999–2002 (visit 9), 2003–2005 (visit 10), and 2007–2009 (visit 11), participants completed a questionnaire in which they reported how many hours they spent sleeping and napping. Self-reported nightly sleeping hours was used for analysis. To maximize stability of sleep duration estimates, values from the 6 assessments were averaged into 3 time-points, grouped according to proximity and strength of pairwise correlations (Table S1; *r* = 0.66–0.70, *p* < 0.001): between visits 4 and 7 (1984–1996; approximately 25 years before MRI), visits 8 and 9 (1997–2002; approximately 15 years before MRI), and visits 10 and 11 (2003–2009; approximately 9 years before MRI).

### 2.3. Health and lifestyle assessment

Education level was assessed at enrollment and converted to years of education. At time of MRI, height and weight were measured with the participant wearing light clothing, without shoes, and used to compute body mass index (BMI, kg/m^2^). History of smoking (never vs former; there were no current smokers), current physical activity (3 or more times per week, yes/no), alcohol consumption (non-drinker/drinker), and medication use were obtained from standard questionnaires. For each visit, participants were coded (yes/no) according to use of medications with central nervous system effects that may impact sleep, including anxiolytic and sedative/hypnotic drugs. Blood pressure was measured in seated, resting participants by a certified nurse, using a regularly calibrated mercury sphygmomanometer. Participants were considered hypertensive if they had an average systolic blood pressure ≥140 mm Hg, diastolic blood pressure ≥90 mm Hg, were taking antihypertensive medication, or reported a diagnosis of hypertension. Depression was assessed using the 21-item Beck depression inventory (BDI) (Beck et al., 1961).

### 2.4. Imaging data acquisition

Imaging data were acquired on a 3.0 Tesla Discovery 750 scanner (GE Healthcare, Milwaukee, WI, USA) at the University of California, San Diego Center for Functional MRI using an 8-channel phased array head coil. The protocol included a 3-plane localizer; a sagittal 3D fast spoiled gradient echo T_1_-weighted structural scan optimized for maximum tissue contrast (TE = 3.2 ms, TR = 8.1 ms, inversion time = 600 ms, flip angle = 8°, FOV = 256 × 256 mm, matrix = 256 × 192, slice thickness = 1.2 mm, resampled to a 1 × 1 × 1.2 mm resolution, scan time 8:27); and an axial 2D single-shot pulsed-field gradient spin-echo echo-planar diffusion-weighted sequence (45 gradient directions, b-values = 500, 1500, 4000 s/mm2, one b = 0 volume and 15 gradient directions for each non-zero b-value; TE = 80.6 ms, TR = 7 s, FOV = 240 × 240 mm, matrix = 96 × 96, slice thickness = 2.5 mm, resampled to a 1.875 × 1.875 × 2.5 mm resolution, scan time 6:34).

### 2.5. Data processing

Structural and diffusion MRI data were processed using FreeSurfer (http://surfer.nmr.mgh.harvard.edu) and in-house software, as previously described (Hagler et al., 2019). Briefly, an iterative Eddy current correction (ECC) approach corrected for distortions in the phase-encode direction of each diffusion MRI frame, whereby displacements are modeled as a function of spatial location, gradient orientation and gradient strength (Hagler et al., 2019). Each diffusion MRI frame was rigid-body registered to the corresponding volume generated by the robust post-ECC tensor fit to correct for head motion (Holland et al., 2010; Jovicich et al., 2006; Zhuang et al., 2006). To correct for geometric and intensity distortions caused by magnetic field inhomogeneities, b = 0 images acquired in opposite phase encoding directions were aligned using nonlinear registration and the resulting displacement volume was used to correct subsequent diffusion weighted images. Tissue boundaries of gray matter (GM), WM, and CSF were identified on T_1_-weighted structural images using FreeSurfer and subcortical regions were automatically segmented according to a subcortical atlas (Fischl et al., 2002). Diffusion MRI data were rigid-body registered to T_1_ images (Hagler et al., 2019).

### 2.6. Computation of RSI metrics

A linear estimation approach leveraging the multi-shell diffusion MRI acquisition was used to model relative fractions of restricted, hindered, and free water diffusion within single voxels, using separate fiber orientation density functions, modelled as fourth order spherical harmonic functions. RSI metrics of interest included restricted isotropic (RI), neurite density (ND), hindered isotropic (HI), and isotropic free water (IF) diffusion (Hagler et al., 2019; White et al., 2013). RI measures non-oriented restricted diffusion and reflects intracellular diffusion within cell bodies. ND measures restricted anisotropic (oriented) diffusion, accounting for crossing fibers, and thus reflects diffusion within neurites (axons and dendrites). IF estimates diffusion in interstitial or cerebrospinal fluid. Thus, the reductions in RI and ND and increases in IF, which have been previously documented in association with mild cognitive impairment (Reas et al., 2017), cognitive aging (Reas et al., 2020, 2021a) vascular dysfunction (Parada et al., 2022), and dietary factors (Tsiknia et al., 2022), are presumed to reflect cytoarchitectural changes including cell shrinkage or death, demyelination, or axon or dendrite loss. Finally, HI presumably reflects diffusion within the extracellular space or within large cell bodies, and has been shown to be reduced with age and poor cognition (Reas et al., 2020, 2021a), but elevated with vascular dysfunction (Parada et al., 2022).

Fifteen WM tracts were derived using a probabilistic atlas-based tract segmentation method, whereby each voxel is assigned a probability value representing the likelihood that it belongs to a particular tract. Probability maps were then used to compute weighted averages for RI, ND, and IF in each tract (Hagler et al., 2009). Since WM is poorly characterized by the hindered fraction, HI was not examined in fiber tracts (White et al., 2013). Voxels containing primarily GM or CSF were excluded from WM tracts (Fischl et al., 2002). Global WM measures were calculated as the mean across all tracts. All RSI metrics were measured within 5 subcortical regions (thalamus, putamen, caudate, hippocampus, and amygdala), and along a vertex-wise map of the cortical GM surface. To minimize partial volume effects for cortical surface-based analyses, RSI metrics were sampled with linear interpolation from 0.8 to 2.0 mm from the GM/WM boundary along the normal to the cortical ribbon and combined using a weighted average based on the proportion of GM in each voxel. RSI cortical surface maps were registered to common space and smoothed with a FWHM 10 mm kernel.

### 2.7. Statistical analysis

Associations of sleep quality (PSQI) and sleep duration with demographic, health, and lifestyle variables were assessed with Pearson’s correlations for continuous variables and ANOVA for categorical variables.

To examine associations of sleep quality with WM and subcortical microstructure, linear regressions were computed for each RSI metric within each fiber tract or subcortical region, with the RSI metric as the dependent variable. Regressions were conducted first with PSQI as the predictor to assess linear associations, and subsequently with PSQI^2^ added to assess nonlinear associations. Base models covaried for age and sex, and secondary models further adjusted for education, smoking, alcohol intake, physical activity, hypertension, BMI, and BDI, with all covariates measured at time of MRI. Sleep medication use was not included as a covariate because it is a PSQI component. For any RSI measure demonstrating a significant linear or non-linear effect of PSQI, regressions were conducted to identify the PSQI components(s) that most strongly contributed to the association. Regressions included the RSI metric as the dependent variable and all covariates listed above for fully adjusted models, along with each of the 7 PSQI components and their squared values entered as stepwise predictors.

To assess associations of prior sleep duration with brain microstructure in later life, RSI measures were examined according to mean sleep duration at 3 time-points (25, 15, and 9 years) prior to MRI. Regressions were computed, as described for sleep quality, with linear and squared terms for sleep duration. Base models adjusted for age at MRI and sex. Secondary models included all covariates used for analyses of PSQI plus sleep medication use at time of sleep assessment.

Vertex-wise linear regressions examined associations of PSQI and sleep duration with cortical GM microstructure, adjusted for age at MRI and sex (projected onto the *fsaverage* template). Models assessed both linear and non-linear terms for sleep quality or duration.

To examine sex differences in associations between sleep measures (quality or duration) and microstructure, regressions were conducted as described above, with additional terms for the interaction between sex and the sleep measure, and between sex and the squared sleep measure, computed as the product of mean-centered sex and sleep measures to reduce multicollinearity among predictors. Significant interactions were followed by sex-stratified analyses.

For comparison of microstructure with morphometric MRI measures, analyses for PSQI and sleep duration were also conducted on WM volume, subcortical volume, and cortical thickness. Volumetric measures were corrected for intracranial volume using the residual after linear regression.

Analyses were performed in FreeSurfer version 6.0 and SPSS version 28.0 (IBM Corp, Armonk, NY, USA). Significance was set to *p* < 0.05. Analyses were adjusted with Bonferroni correction, using a threshold of *p* < 0.003 for PSQI models adjusted for comparison across 15 fiber tracts and *p* < 0.01 for comparison across 5 subcortical regions. Analyses of sleep duration were further adjusted for comparison across 3 timepoints, using significance thresholds of *p* < 0.017 for global white matter, *p* < 0.001 for fiber tracts, and *p* < 0.003 for subcortical regions. Cortical thickness analyses were corrected with the false discovery rate (FDR) method.

## 3. Results

### 3.1. Participant characteristics

Participant characteristics according to sleep quality (*N* = 117) and sleep duration (*N* = 143) are presented in Table 1. Higher PSQI scores (measured an average of 6.3 ± 0.7 years prior to MRI) correlated with shorter sleep duration (9 years before MRI, *p* = 0.005), more frequent use of sleep medication (*p* = 0.009), and higher BDI (*p* < 0.001). Shorter sleep duration was associated with higher education level (*p* = 0.03). Mean sleep length was relatively stable over visits and increased by 2.4 minutes from the assessments 25–9 years prior to MRI. Sleep medication use increased over time, with 6%, 9%, and 15% of subjects reporting use of sleep medications at the assessments 25, 15, and 9 years before MRI, respectively.

### 3.2. Associations of sleep quality and duration with brain microstructure

**Fig. 1.**
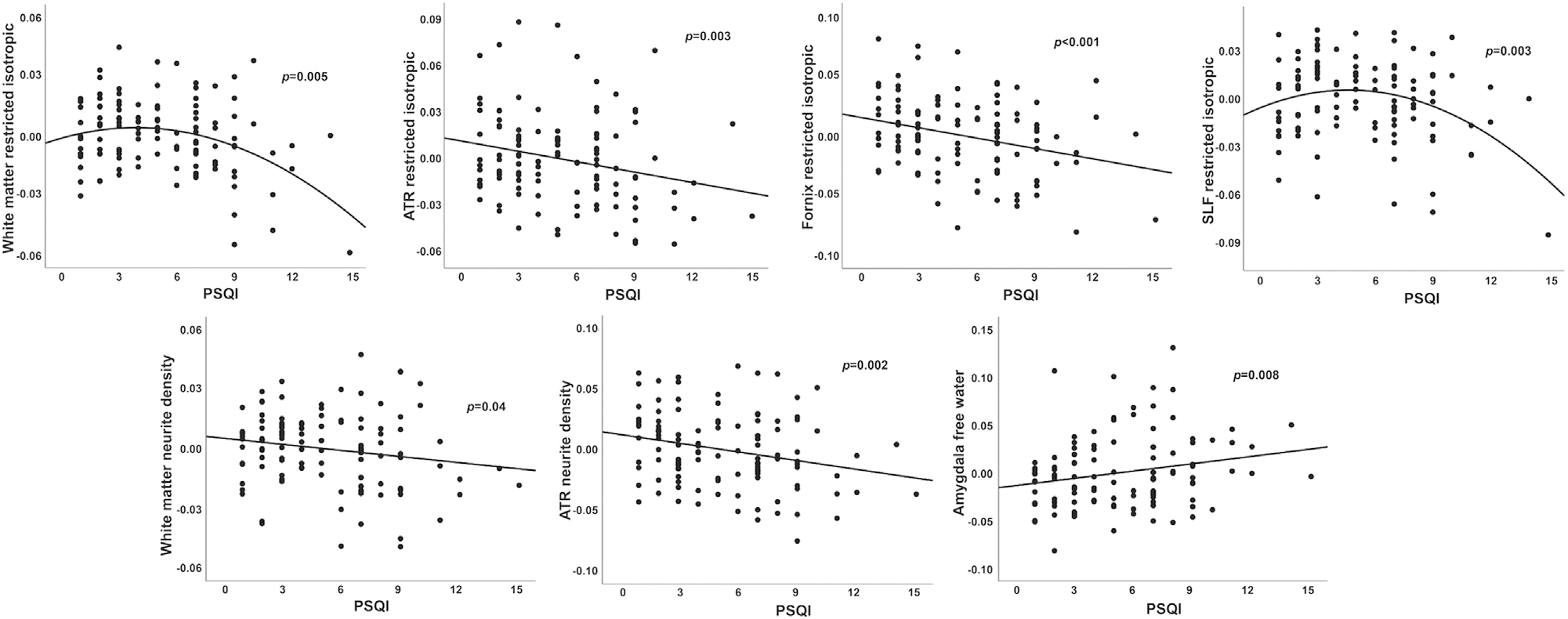
Associations between sleep quality and brain microstructure. Poor sleep quality (higher PSQI) correlated with lower WM restricted isotropic diffusion (intracellular) and neurite density, and with higher amygdala free water. Values are residuals, adjusted for age, sex, education, alcohol consumption, smoking, physical activity, hypertension, body mass index, and Beck depression inventory. Abbreviations: ATR, anterior thalamic radiation; PSQI, Pittsburg sleep quality index; SLF, superior longitudinal fasciculus.

In base models (adjusted for age and sex, Bonferroni corrected), worse sleep quality was associated with lower RI in global WM and within several WM tracts (corticospinal tract (CST), inferior longitudinal fasciculus (ILF), superior longitudinal fasciculus (SLF), superior corticostriatal (SCS)), and with lower ILF ND. In models further adjusted for education, alcohol consumption, smoking, physical activity, hypertension, BMI, and depression (Tables S2 and S3), PSQI was linearly associated with RI in the fornix and anterior thalamic radiation (ATR), global white matter and ATR ND, and amygdala IF. Non-linear (PSQI^2^) associations were present for RI across all WM and in the SLF. As shown in Fig. 1, worse sleep quality was associated with lower RI and ND and with higher IF, with non-linear relationships reflecting stronger associations with worse sleep quality.

When each of the linear and quadratic PSQI components were considered as independent predictors in place of the composite PSQI score, a combination of quadratic subjective sleep quality, quadratic sleep medication use, linear daytime dysfunction, and linear and quadratic sleep efficiency best predicted fiber tract RI and ND, and amygdala IF (Table S4). Quadratic sleep disturbances additionally predicted amygdala IF.

Neither the linear nor quadratic sleep duration components of the PSQI were strong predictors of brain microstructure relative to other components. Therefore, to evaluate sleep duration at earlier life periods, when coexisting age-related sleep disturbances would be less prominent, we next examined associations between sleep duration and microstructure 9, 15, and 25 years before MRI. Linear associations between microstructure and prior sleep duration were modest at all time-points, with no associations reaching significance after Bonferroni correction for multiple comparisons (Tables S5–S7). Inclusion of squared terms for sleep duration did not significantly improve model fit.

### 3.3. Sex differences in associations between sleep patterns and brain microstructure

**Fig. 2.**
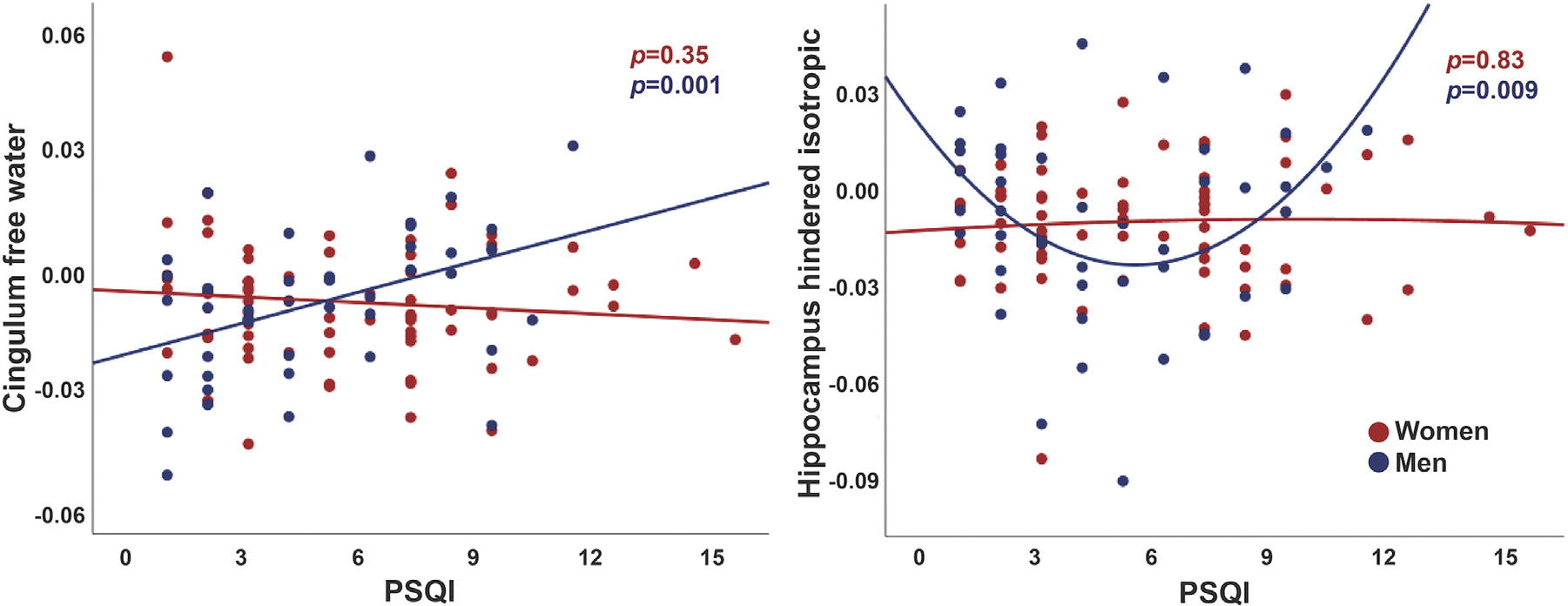
Sex differences in associations between sleep quality and brain microstructure. Sleep quality (PSQI, Pittsburg sleep quality index) was associated with cingulum free water and hippocampal hindered isotropic diffusion for men only. Values are residuals, adjusted for age, education, alcohol consumption, smoking, physical activity, hypertension, body mass index, and Beck depression inventory.

Women had fewer years of education, lower BMIs, and were less likely to be married than men (*p* ≤ 0.001, Table S8), but there were no sex differences in PSQI scores or its subcomponents, or in sleep duration at any assessment. When base regression models included terms for the interaction between sex and sleep quality, sex interactions were significant with linear PSQI for cingulum IF (*p* < 0.001) and hippocampal HI (*p* = 0.005), and with PSQI^2^ for forceps major ND (*p* = 0.002) and hippocampal HI (*p* = 0.003). Interactions for cingulum IF (*p* < 0.001) and hippocampal HI (*p* = 0.002) remained significant after full adjustment. Sex-stratified regressions revealed associations only among men between higher PSQI and higher cingulum IF, and between PSQI^2^ and hippocampal HI, reflecting higher HI with the lowest and highest sleep quality ratings (Fig. 2).

**Fig. 3.**
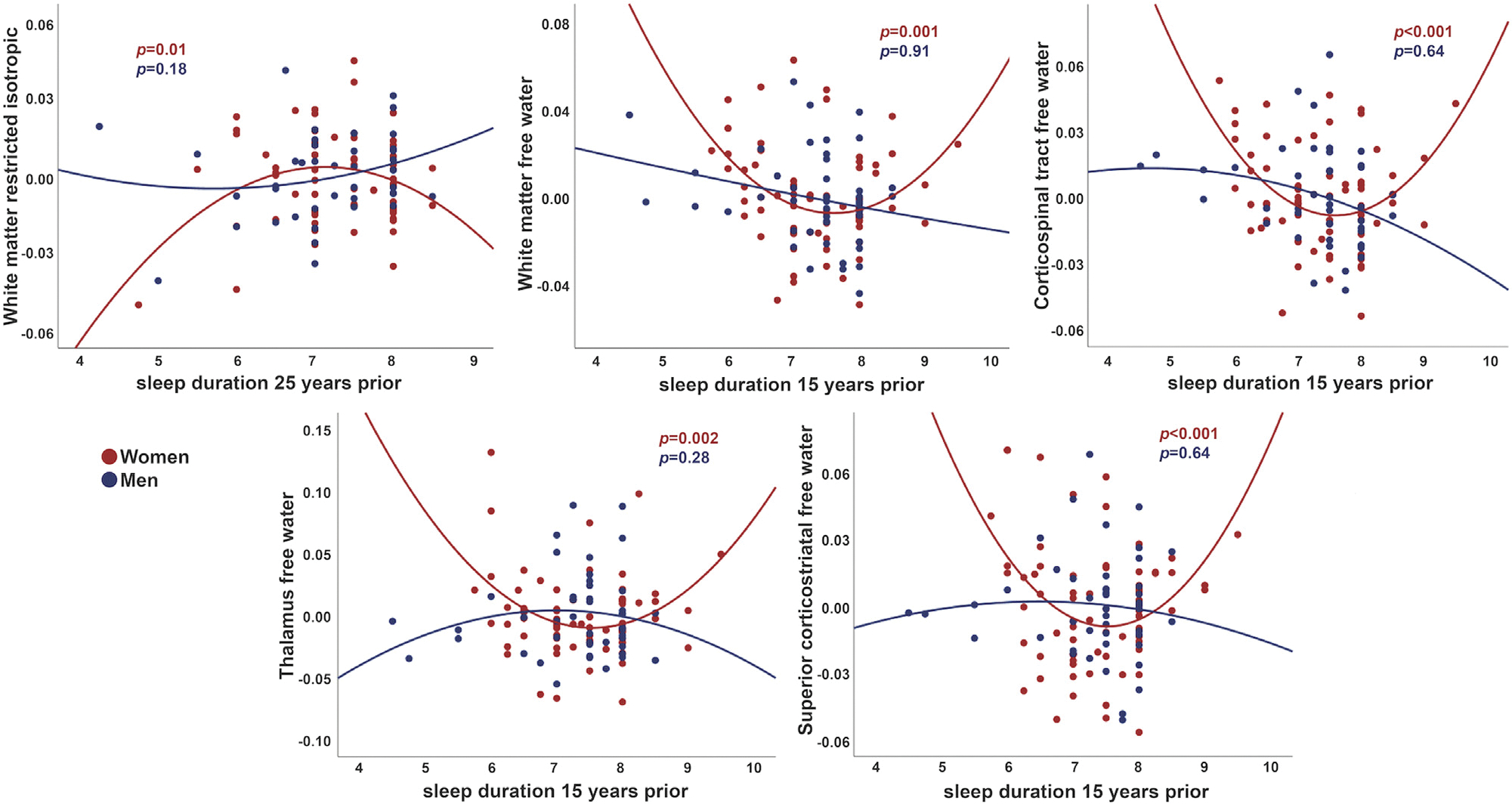
Sex differences in associations between prior sleep duration and brain microstructure. Sleep duration 25 and 15 years prior to MRI was associated with white matter and thalamus microstructure for women only. Values are residuals, adjusted for age, education, alcohol consumption, smoking, physical activity, hypertension, body mass index, Beck depression inventory, and sleep medication.

In base models, sex interactions were present with quadratic sleep duration 25 years prior to MRI for global WM RI (*p* = 0.01), and with linear and quadratic terms for sleep duration 15 years prior to MRI for IF in global WM (*p* < 0.001) and within the CST (*p* < 0.001), ATR (*p* < 0.001), SCS (*p* < 0.001), thalamus (*p* < 0.001), and putamen (*p* < 0.01). Interactions remained significant after full adjustment for quadratic sleep duration for global WM RI (*p* = 0.015, 25 years prior to MRI) as well as for IF in global WM (*p* = 0.005), the CST (*p* < 0.001), SCS (*p* < 0.001), and thalamus (*p* < 0.001) (15 years before MRI). In contrast to results for sleep quality, sex-stratified regressions revealed significant associations for women only, reflecting lower RI and higher IF with both shorter and longer sleep durations (Fig. 3). Sex did not interact with sleep duration 9 years before MRI.

### 3.4. Sleep patterns are not associated with brain volume or thickness

To assess whether prior sleep patterns are associated with morphometric brain measures, we examined associations of sleep quality and duration with white matter volume, subcortical volume, and cortical gray matter thickness. There were no significant associations between linear or quadratic sleep quality or duration and morphometric MRI measures (Table S9). Sex did not interact with sleep quality or duration for any RSI measure in cortical gray matter or for any morphometric measure.

## 4. Discussion

In this study of community-dwelling older adults without dementia, worse sleep quality predicted RSI measures reflecting abnormal brain cytoarchitecture after a 6-year follow-up, independently of other health and lifestyle factors, with subjective sleep quality, sleep medication use, sleep efficiency, daytime dysfunction, and sleep disturbances demonstrating the strongest associations with WM and subcortical microstructure. Sex interacted with sleep quality, such that men exhibited stronger associations between poor sleep quality and brain microstructure than women. Although sleep duration did not predict brain microstructure across the whole cohort, sex-interaction analyses revealed stronger associations between short or long sleep duration 25 and 15 years prior to MRI and abnormal brain microstructure only among women.

Worse sleep quality was associated with lower global WM RI and ND, indicating reduced intracellular diffusion that likely reflects axon loss or demyelination, and with higher amygdala free water, reflecting expansion of the CSF space. The presence of both linear and non-linear associations suggests that deleterious effects of impaired sleep on brain health are magnified among those with more severe sleep disturbances. In an overlapping sample, we previously found correlations of lower RI and ND and higher free water with older chronological age, yet here, effects of sleep quality exceeded those attributable to age, suggesting that poor sleep quality may compound axonal damage and neuronal shrinkage or dystrophy typically seen in normal aging (Reas et al., 2020). These results extend evidence that poor sleep quality is associated with brain atrophy and WM hyperintensities (Ramos et al., 2014; Sexton et al., 2014), to suggest that sleep disturbances may promote subtle cytoarchitectural changes preceding widespread neurodegeneration or WM pathology. In further support of this interpretation, sleep patterns did not predict cortical thinning or loss of WM or subcortical volume. Our findings are at odds with prior diffusion tensor imaging (DTI) studies reporting either weak (Kocevska et al., 2019) or no associations (Sexton et al., 2017) between poor sleep quality and abnormal WM microstructure, as indexed by decreased fractional anisotropy and increased diffusivity. These discrepancies may be explained by the improved characterization of cytoarchitectural organization afforded by RSI compared to DTI (Reas et al., 2020), which is equipped to better isolate the intra-axonal fraction by excluding contributions from CSF and interstitial fluid and accounting for fiber complexity.

Interaction analyses revealed stronger associations between sleep quality and brain microstructure for men than for women. Specifically, we observed a linear association between worse sleep quality and higher cingulum IF, only in men. Higher IF reflects a greater contribution from the extracellular compartment to the diffusion signal, which in white matter may accompany axon loss or demyelination. Additionally, we found a non-linear association between sleep quality and hippocampus HI only among men, with higher HI for the lowest and highest PSQI scores. Because HI can reflect both intracellular diffusion within large cell bodies, and extracellular diffusion, the HI signal may be dominated by distinct cytoarchitectural properties in different contexts; for instance, high HI could perhaps be indicative of preservation of large cell bodies with high quality sleep, but an increase in the extracellular space with severely impaired sleep quality. Although prior work suggests sex-specific patterns of sleep problems, including more severe objective sleep disturbances among men yet worse subjective sleep complaints in women (Mong and Cusmano, 2016), we observed no sex differences in overall sleep quality or its subcomponents. Prior evidence suggests that women are more likely to report worse sleep quality in the absence of objective sleep disturbances (van den Berg et al., 2009), which may have weakened the association between self-reported sleep quality and brain microstructure among women in our study. Future studies assessing sleep quality using both subjective and objective measures will be necessary to determine whether impaired sleep is more consequential to cytoarchitectural integrity among older men.

Although sleep duration did not predict brain microstructure in the whole cohort, we observed non-linear effects of sleep duration reported 25 and 15 years prior to MRI on WM and thalamus microstructure among women only. Women reporting extreme sleep durations had lower WM RI and higher thalamus and WM IF, with optimal microstructure (highest RI and lowest IF) for 7–8 hours nightly sleep. This range aligns well with prior studies reporting non-linear associations of sleep duration with brain and cognitive aging that similarly identified an optimal nightly target of 7–8 hours (Li et al., 2022; Sabia et al., 2021; Yaffe et al., 2014). It is unclear from these observational findings whether the double-dissociation between sex differences for sleep quality and sleep duration reflects differential effects of sleep quality and duration on brain health for men or women, or may be partially influenced by differences in the timing of the sleep measurements. Because there were greater proportions of perimenopausal women at sleep duration assessments 25 and 15 years prior to MRI (mean age: 54.4 ± 7.1 and 62.9 ± 7.2 years, respectively), compared to sleep duration assessments 9 years prior to MRI (68.0 ± 7.6 years) or sleep quality assessments 6 years prior to MRI (mean age: 70.2 ± 8.0 years), hormonal changes may have exacerbated sleep disruptions during these earlier periods and contributed to more pronounced effects of inadequate or prolonged sleep on brain aging trajectories. The menopausal transition is associated with a decline in sleep duration and actigraphy-derived measures of sleep quality, as well as an increase in the prevalence of obstructive sleep apnea (Lampio et al., 2017). Therefore, despite similar reports of sleep duration for men and women around midlife, hot flashes, vasomotor symptoms, and other menopause-related sleep disturbances might make women particularly vulnerable to the deleterious effects of inadequate sleep. Considering the apparently complex orchestration of sleep circuitry by sex hormones (Lord et al., 2014) and link-age with sex chromosomes (Ehlen et al., 2013), further examination of neuroendocrine or genetic pathways that may differentially mediate neurophysiological responses to sleep disruptions for aging men and women is warranted.

Regional analyses identified the strongest correlations between sleep measures and cytoarchitecture in subcortical and limbic structures including the thalamus, amygdala, hippocampus, and fornix, and in projection and association fibers (cingulum, CST, ATR, SCS, ILF and SLF). The thalamus receives efferent signals from sleep-controlling hubs such as the hypothalamus and brainstem, and sends widespread afferent projections, making it a crucial component of sleep-regulating neural circuitry (Gent et al., 2018) that regulates circadian rhythms and sleep homeostasis (Gent et al., 2018; Krishnan et al., 2018; Qiu et al., 2010; Vetrivelan et al., 2010). Furthermore, the thalamus orchestrates the production of sleep spindles and slow-wave oscillations in the striatum, hippocampus and neocortex (Krishnan et al., 2018), which are essential for facilitating sleep-dependent memory consolidation (Fogel and Smith, 2011). Microstructural abnormalities in limbic structures such as the amygdala, hippocampus, and fornix may disturb communication between sleep circuitry and memory consolidation networks, possibly explaining well-documented links between poor sleep quality and memory decline (Cruz et al., 2019). Furthermore, ATR and SCS fibers projecting from the thalamus and striatum to frontal neocortex may facilitate the link between sleep-regulating circuitry (Vetrivelan et al., 2010) and frontal regions involved in higher-order cognitive functions such as executive function, for which sleep plays a crucial supportive role by preserving the structural integrity of frontal networks (Holanda and de Almondes, 2016).

Our study has some limitations. Sleep quality was based on self-report rather than actigraphy, although evidence for strong correlations between subjective and objective measures of sleep quality in older adults minimizes this concern (Gaiduk et al., 2021). Furthermore, we did not account for the possible influence of OSA, which influences sleep quality and may have sex-dependent effects on cognitive impairment (Snyder and Cunningham, 2018), or other unmeasured confounders. Because imaging was conducted at a single time-point, we were unable to assess effects of sleep on longitudinal brain changes. Finally, our sample size and composition of predominantly highly educated non-Hispanic White participants may have limited power to detect differences by sex or socioeconomic status, which limits generalizability of our findings, but likely reduced the influence of confounding socioeconomic factors.

## 5. Conclusions

Our findings extend prior evidence implicating poor sleep quality in age-related brain atrophy to demonstrate that sleep disturbances are associated with subtle microstructural brain injury even in the absence of detectable volume loss or cortical thinning, and these effects might be more pronounced for older men. In women, inadequate or excessive sleep experienced as early as midlife may modify brain aging trajectories to elicit microstructural abnormalities that manifest decades later, highlighting the clinical urgency of developing strategies to optimize sleep behaviors early in the aging process. Future studies examining sleep behaviors relative to longitudinal changes in brain microstructure, and their modification by health and lifestyle factors, will help to guide precision medicine recommendations that will maximize healthy brain aging.

## Supplementary Material

Supplementary material

## Figures and Tables

**Table 1:** Participant characteristics at MRI and associations with sleep quality (PSQI) and with sleep duration approximately 9 years prior to MRI

Variable	Mean ± SD or *N*(%)	Association with PSQI	Association with sleep duration

Age at MRI (years)	76.6 ± 8.0	*r* = −0.05, *p* = 0.58	*r* = −0.13, *p* = 0.13
Sex (women)	*N* = 71 (61%)	*F*(1,115) = 1.10, *p* = 0.30	*F*(1,141) = 0.48, *p* = 0.49
PSQI (6 years prior)	5.3 ± 3.2	NA	***r* = −0.26, *p* = 0.005**
Hours asleep (9 years prior)	7.3 ± 0.9	***r* = −0.26, *p* = 0.005**	NA
Education (years)	14.9 ± 2.1	*r* = −0.03, *p* = 0.78	***r* = −0.18, *p* = 0.03**
Sleep medication use	*N* = 26 (23%)	***F*(1,113) = 7.07, *p* = 0.009**	*F*(1,141) = 0.00, *p* = 0.95
Hypertension	*N* = 70 (60%)	*F*(1,115) = 0.06, *p* = 0.81	*F*(1,141) = 0.34, *p* = 0.56
*APOE* (*ε*4-carrier)	*N* = 27 (24%)	*F*(1,110) = 2.41, *p* = 0.12	*F*(1,133) = 0.01, *p* = 0.91
Exercise (3+ times/week)	*N* = 90 (78%)	*F*(1,114) = 0.18, *p* = 0.67	*F*(1,140) = 0.06, *p* = 0.81
Body mass index (BMI, kg/m^2^)	25.7 ± 3.7	*r* = 0.05, *p* = 0.60	*r* = 0.09, *p* = 0.28
Smoking (ever)	*N* = 51 (44%)	*F*(1,114) = 0.02, *p* = 0.89	*F*(1,140) = 0.03, *p* = 0.87
Alcohol (current drinker)	*N* = 103 (89%)	*F*(1,114) = 1.87, *p* = 0.17	*F*(1,140) = 0.07, *p* = 0.80
Beck depression inventory	4.0 ± 3.6	***r* = 0.49, *p* < 0.001**	*r* = −0.09, *p* = 0.30
Marital status (married)	*N* = 90 (78%)	*F*(1,114) = 0.59, *p* = 0.44	*F*(1,140) = 0.40, *p* = 0.53

Sleep medication use is at any sleep assessment for PSQI, and 9 years before MRI for sleep duration. BMI is adjusted for sex. **BOLD** = *p* < 0.05
