# Supplementary material for: Sleep quality and sleep duration predict brain microstructure among community-dwelling older adults
Source: Neurobiol Aging. Author manuscript; Available in PMC 2023 May 1. (PMC10115563; doi:10.1016/j.neurobiolaging.2023.02.001)
Supplement: Supplementary material [file NIHMS1888326-supplement-Supplementary_material.docx]

**Supplemental Material: Sleep quality and sleep duration predict brain microstructure among community-dwelling older adults**

Amaryllis A. Tsiknia, MS ^a^, Humberto Parada Jr, PhD ^b^, Sarah J. Banks, PhD ^a^, Emilie T. Reas, PhD ^a^*

^a^ Department of Neurosciences, University of California, San Diego

^b^ Division of Epidemiology and Biostatistics, San Diego State University

*Address correspondence to Dr. Emilie T. Reas, Department of Neurosciences, Mail code 0841, UCSD, 9500 Gilman Dr., La Jolla, CA 92093-0841; 1-858-822-1518; [ereas@ucsd.edu](about:blank)

**Table S1**. Pearson correlation coefficients between sleep duration estimates at each visit (top right diagonal) and available *N* for the respective correlation (bottom left diagonal).

|  | **Visit 4**  **(1984-87)** | **Visit 7**  **(1992-96)** | **Visit 8**  **(1997-99)** | **Visit 9**  **(1999-2002)** | **Visit 10**  **(2003-06)** | **Visit 11**  **(2007-09)** |
| --- | --- | --- | --- | --- | --- | --- |
| **Visit 4** | - | **0.68***** | 0.52*** | 0.35* | 0.37** | 0.46** |
| **Visit 7** | **47** | - | 0.61*** | 0.64*** | 0.60*** | 0.50*** |
| **Visit 8** | 41 | 99 | - | **0.66***** | 0.53*** | 0.54*** |
| **Visit 9** | 45 | 85 | **101** | - | 0.58*** | 0.65*** |
| **Visit 10** | 53 | 117 | 79 | 98 | - | **0.70***** |
| **Visit 11** | 42 | 90 | 101 | 76 | **102** | - |

Bolded values indicate correlation coefficients and available *N* used for the three averaged sleep duration measures. * *p*<0.05, ** *p*<0.01, *** *p*<0.001

**Table S2**. Standardized beta-estimates from fully adjusted linear regression models for each RSI metric associated with PSQI scores.

| **Predictor** | **WM RI** | **Fornix RI** | **ATR RI** | **SLF RI** | **WM ND** | **ATR ND** | **Amygdala IF** |
| --- | --- | --- | --- | --- | --- | --- | --- |
| **Age** | -0.580*** | -0.542*** | -0.589*** | -0.409*** | -0.463*** | -0.478*** | 0.326*** |
| **Sex** | -0.001 | -0.103 | -0.009 | -0.063 | 0.004 | 0.025 | 0.190 |
| **Education** | 0.085 | -0.036 | 0.089 | 0.025 | 0.113 | 0.026 | -0.088 |
| **Alcohol** | -0.052 | 0.069 | 0.050 | -0.072 | -0.162 | 0.059 | -0.034 |
| **Smoking** | -0.143* | -0.112 | -0.089 | -0.148 | -0.125 | -0.091 | -0.141 |
| **Physical activity** | 0.088 | 0.029 | 0.097 | 0.076 | 0.106 | 0.059 | 0.028 |
| **Hypertension** | -0.068 | -0.132 | -0.050 | -0.048 | -0.038 | 0.037 | 0.125 |
| **BMI** | -0.069 | -0.118 | -0.065 | -0.116 | -0.194* | -0.097 | 0.148 |
| **BDI** | 0.286*** | 0.302*** | 0.206* | 0.279** | 0.233* | 0.268** | -0.171 |
| **PSQI** | 0.369 | -0.310*** | -0.265** | 0.568* | -0.200* | -0.301** | 0.272** |
| **PSQI^2^** | -0.704** | - | - | -0.867** | - | - | - |

ATR, anterior thalamic radiation; BDI, Beck depression inventory; BMI, body mass index; IF, isotropic free water; ND, neurite density; PSQI, Pittsburg sleep quality index; RI, restricted isotropic, SLF, superior longitudinal fasciculus; WM, white matter. * *p*<0.05, ** *p*<0.01, *** *p*<0.001

**Table S3**. Standardized beta estimates for fully adjusted regression models assessing associations between sleep quality (PSQI) and brain microstructure.

|  |  | **RI** | | **ND** | | **IF** | | **HI** | |
| --- | --- | --- | --- | --- | --- | --- | --- | --- | --- |
| **Region** | **Predictor** | **Beta** | ***p*-value** | **Beta** | ***p*-value** | **Beta** | ***p*-value** | **Beta** | ***p*-value** |
| All fibers | PSQI | 0.37 | 0.14 | -0.20 | **0.04** | 0.15 | 0.09 |  |  |
|  | PSQI^2^ | -0.70 | **0.005** |  |  |  |  |  |  |
| Fornix | PSQI | -0.31 | **<0.001** | -0.20 | 0.02 | 0.22 | 0.004 |  |  |
| Cingulum | PSQI | 0.32 | 0.24 | -0.04 | 0.72 | 0.14 | 0.19 |  |  |
|  | PSQI^2^ | -0.61 | 0.03 |  |  |  |  |  |  |
| Parahippocampal cingulum | PSQI | -0.18 | 0.06 | -0.12 | 0.27 | 0.09 | 0.43 |  |  |
| CST | PSQI | 0.40 | 0.15 | -0.02 | 0.84 | 0.14 | 0.12 |  |  |
|  | PSQI^2^ | -0.73 | 0.009 |  |  |  |  |  |  |
| ATR | PSQI | -0.26 | **0.003** | -0.30 | **0.002** | 0.19 | 0.03 |  |  |
| Uncinate | PSQI | -0.22 | 0.01 | -0.14 | 0.16 | 0.04 | 0.67 |  |  |
| Forceps major | PSQI | 0.47 | 0.12 | -0.10 | 0.34 | 0.11 | 0.23 |  |  |
|  | PSQI^2^ | -0.67 | 0.03 |  |  |  |  |  |  |
| Forceps minor | PSQI | -0.25 | 0.006 | -0.19 | 0.04 | 0.19 | 0.06 |  |  |
| Corpus callosum | PSQI | 0.42 | 0.11 | -0.19 | 0.04 | 0.19 | 0.04 |  |  |
|  | PSQI^2^ | -0.71 | 0.007 |  |  |  |  |  |  |
| ILF | PSQI | 0.49 | 0.07 | 0.50 | 0.10 | 0.14 | 0.13 |  |  |
|  | PSQI^2^ | -0.76 | 0.007 | -0.73 | 0.02 |  |  |  |  |
| IFO | PSQI | 0.43 | 0.11 | -0.11 | 0.23 | 0.09 | 0.27 |  |  |
|  | PSQI^2^ | -0.71 | 0.009 |  |  |  |  |  |  |
| SLF | PSQI | 0.57 | 0.05 | 0.42 | 0.20 | 0.04 | 0.73 |  |  |
|  | PSQI^2^ | -0.87 | **0.003** | -0.67 | 0.04 |  |  |  |  |
| SCS | PSQI | 0.35 | 0.20 | -0.01 | 0.94 | 0.06 | 0.56 |  |  |
|  | PSQI^2^ | -0.69 | 0.01 |  |  |  |  |  |  |
| SIFC | PSQI | -0.24 | 0.005 | 0.03 | 0.78 | 0.10 | 0.32 |  |  |
| IFSF | PSQI | 0.39 | 0.16 | -0.17 | 0.11 | 0.11 | 0.31 |  |  |
|  | PSQI^2^ | -0.73 | 0.008 |  |  |  |  |  |  |
| Thalamus | PSQI | -0.17 | 0.13 | -0.15 | 0.17 | 0.05 | 0.63 | 0.13 | 0.24 |
| Caudate | PSQI | -0.20 | 0.08 | 0.02 | 0.81 | 0.08 | 0.43 | 0.08 | 0.40 |
| Putamen | PSQI | -0.22 | 0.06 | -0.17 | 0.15 | 0.05 | 0.68 | 0.16 | 0.17 |
| Hippocampus | PSQI | -0.15 | 0.12 | -0.15 | 0.13 | 0.02 | 0.87 | 0.03 | 0.77 |
| Amygdala | PSQI | -0.13 | 0.18 | -0.03 | 0.76 | 0.27 | **0.008** | -0.18 | 0.11 |
|  |  |  |  |  |  |  |  |  |  |

Beta estimates are for models including only linear PSQI, unless addition of PSQI^2^ significantly (*p*<0.05) improved model fit. Models are adjusted for age, sex, education, alcohol consumption, smoking, physical activity, hypertension, BMI, and depression. HI was not computed in white matter tracts. *P*-values are unadjusted. Beta values reaching significance after multiple comparisons correction are bolded (*p*<0.05 for all fibers, *p*<0.003 for fiber tracts, *p*<0.01 for subcortical regions). ATR, anterior thalamic radiation; CST, corticospinal tract; HI, hindered isotropic; IF, isotropic free water; IFSF, inferior frontal superior frontal; ILF, inferior longitudinal fasciculus, IFO, inferior frontal occipital; ND, neurite density; PSQI, Pittsburg sleep quality index; RI, restricted isotropic, SCS, superior corticostriatal, SIFC, superior inferior frontal cortex; SLF, superior longitudinal fasciculus

**Table S4**. Standardized beta-estimates from fully adjusted linear regression models for each RSI metric associated with PSQI scores, with PSQI component scores (linear and squared) entered stepwise as predictors.

| **Predictor** | **WM RI** | **Fornix RI** | **ATR RI** | **SLF RI** | **WM ND** | **ATR ND** | **Amygdala IF** |
| --- | --- | --- | --- | --- | --- | --- | --- |
| **Age** | -0.587*** | -0.522*** | -0.619*** | -0.413*** | -0.515*** | -0.506*** | 0.034*** |
| **Sex** | 0.027 | -0.102 | 0.031 | -0.030 | 0.028 | 0.065 | 0.196* |
| **Education** | 0.055 | -0.032 | 0.057 | -0.007 | 0.060 | -0.007 | -0.113 |
| **Alcohol** | -0.047 | 0.038 | 0.026 | -0.061 | -0.175* | 0.033 | 0.000 |
| **Smoking** | -0.141 | -0.126 | -0.093 | -0.148 | -0.101 | -0.089 | -0.176* |
| **Physical activity** | 0.119 | 0.041 | 0.050 | 0.112 | 0.069 | 0.017 | 0.026 |
| **Hypertension** | -0.115 | -0.164* | -0.089 | -0.092 | -0.078 | 0.002 | 0.113 |
| **BMI** | -0.101 | -0.099 | -0.060 | -0.163 | -0.188* | -0.092 | 0.114 |
| **BDI** | 0.316*** | 0.340*** | 0.263** | 0.310** | 0.215* | 0.293** | -0.255** |
| **Sleep quality^2^** | -0.282*** | *NS* | -0.182* | -0.258** | -0.361*** | -0.204* | *NS* |
| **Sleep efficiency** | *NS* | -0.148* | *NS* | *NS* | *NS* | *NS* | 0.179* |
| **Sleep efficiency^2^** | *NS* | *NS* | *NS* | *NS* | 0.258** | *NS* | *NS* |
| **Sleep disturbances^2^** | *NS* | *NS* | *NS* | *NS* | *NS* | *NS* | 0.243** |
| **Sleep medications^2^** | -0.201** | -0.233** | *NS* | -0.180* | *NS* | *NS* | *NS* |
| **Daytime dysfunction** | *NS* | -0.184* | -0.269*** | *NS* | *NS* | -0.207* | 0.231* |

Only PSQI components that were included in final models are presented; neither linear nor quadratic terms for sleep latency or sleep duration were included in any model. *NS* = not significant in stepwise regressions and not included in final model. ATR, anterior thalamic radiation; BDI, Beck depression inventory; BMI, body mass index, IF, isotropic free water; ND, neurite density; RI, restricted isotropic, SLF, superior longitudinal fasciculus; WM, white matter. * *p*<0.05, ** *p*<0.01, *** *p*<0.001

**Table S5**. Standardized beta estimates for fully adjusted regression models assessing associations between sleep duration 25 years before MRI and brain microstructure.

|  |  | **RI** | | **ND** | | | **IF** | | **HI** | |
| --- | --- | --- | --- | --- | --- | --- | --- | --- | --- | --- |
| **Region** | **Predictor** | **Beta** | ***p*-value** | **Beta** | ***p*-value** | **Beta** | | ***p*-value** | **Beta** | ***p*-value** |
| All fibers | Sleep duration | 0.13 | 0.09 | 0.05 | 0.60 | -0.09 | | 0.27 |  |  |
| Fornix | Sleep duration | 0.17 | 0.02 | 0.07 | 0.31 | -0.11 | | 0.11 |  |  |
| Cingulum | Sleep duration | 0.08 | 0.31 | 0.03 | 0.71 | -0.10 | | 0.27 |  |  |
| Parahippocampal cingulum | Sleep duration | -0.01 | 0.95 | -0.05 | 0.61 | 0.00 | | 0.98 |  |  |
| CST | Sleep duration | 0.19 | 0.02 | 0.13 | 0.13 | -0.13 | | 0.11 |  |  |
| ATR | Sleep duration | 0.07 | 0.38 | 0.02 | 0.81 | -0.10 | | 0.22 |  |  |
| Uncinate | Sleep duration | 0.07 | 0.39 | -0.02 | 0.87 | -0.05 | | 0.62 |  |  |
| Forceps major | Sleep duration | 0.01 | 0.89 | 0.02 | 0.78 | -0.08 | | 0.35 |  |  |
| Forceps minor | Sleep duration | 2.15 | 0.01 | 0.07 | 0.41 | -0.01 | | 0.91 |  |  |
|  | Sleep duration^2^ | -2.10 | 0.02 |  |  |  | |  |  |  |
| Corpus callosum | Sleep duration | 0.11 | 0.16 | 0.07 | 0.37 | -0.08 | | 0.30 |  |  |
| ILF | Sleep duration | 0.07 | 0.42 | 0.08 | 0.37 | -0.13 | | 0.12 |  |  |
| IFO | Sleep duration | 0.07 | 0.40 | 0.00 | 0.98 | -0.02 | | 0.75 |  |  |
| SLF | Sleep duration | 0.19 | 0.03 | 0.06 | 0.56 | -0.09 | | 0.33 |  |  |
| SCS | Sleep duration | 0.18 | 0.03 | -0.01 | 0.90 | -0.04 | | 0.67 |  |  |
| SIFC | Sleep duration | 0.10 | 0.21 | -0.08 | 0.36 | -0.04 | | 0.64 |  |  |
| IFSF | Sleep duration | 0.17 | 0.04 | -0.03 | 0.73 | -0.06 | | 0.53 |  |  |
| Thalamus | Sleep duration | -0.02 | 0.84 | -0.17 | 0.08 | 0.00 | | 1.00 | 0.10 | 0.31 |
| Caudate | Sleep duration | 0.50 | 0.59 | -0.08 | 0.33 | -0.03 | | 0.74 | 0.00 | 0.98 |
| Putamen | Sleep duration | 0.10 | 0.36 | 0.07 | 0.52 | 0.04 | | 0.73 | -0.11 | 0.31 |
| Hippocampus | Sleep duration | -0.01 | 0.92 | 0.02 | 0.79 | -0.13 | | 0.13 | 0.12 | 0.17 |
| Amygdala | Sleep duration | -0.01 | 0.89 | 0.08 | 0.39 | -0.05 | | 0.55 | 0.04 | 0.67 |

Beta estimates are for models including only linear sleep duration, unless including squared sleep duration significantly (*p*<0.05) improved model fit. Models are adjusted for age, sex, education, alcohol consumption, smoking, physical activity, hypertension, BMI, depression, and sleep medication use. HI was not computed in white matter tracts. *P*-values are unadjusted. Linear or squared sleep duration terms did not reach significance after Bonferroni correction for multiple comparisons (*p*<0.017 for all fibers, *p*<0.001 for fiber tracts, *p*<0.003 for subcortical regions). ATR, anterior thalamic radiation; CST, corticospinal tract; HI, hindered isotropic; IF, isotropic free water; IFSF, inferior frontal superior frontal; ILF, inferior longitudinal fasciculus, IFO, inferior frontal occipital; ND, neurite density; RI, restricted isotropic, SCS, superior corticostriatal, SIFC, superior inferior frontal cortex; SLF, superior longitudinal fasciculus

**Table S6**. Standardized beta estimates for fully adjusted regression models assessing associations between sleep duration 15 years before MRI and brain microstructure.

|  |  | **RI** | | | **ND** | | | **IF** | | | **HI** | | |
| --- | --- | --- | --- | --- | --- | --- | --- | --- | --- | --- | --- | --- | --- |
| **Region** | **Predictor** | **Beta** | ***p*-value** | **Beta** | | ***p*-value** | **Beta** | | ***p*-value** | **Beta** | | ***p*-value** |  |
| All fibers | Sleep duration | 0.08 | 0.26 | 0.20 | | 0.02 | -0.17 | | 0.03 |  | |  |  |
| Fornix | Sleep duration | 0.04 | 0.54 | 0.13 | | 0.09 | -0.09 | | 0.20 |  | |  |  |
| Cingulum | Sleep duration | 0.01 | 0.93 | 0.11 | | 0.22 | -0.19 | | 0.05 |  | |  |  |
| Parahippocampal cingulum | Sleep duration | -0.10 | 0.23 | 0.03 | | 0.74 | -0.04 | | 0.68 |  | |  |  |
| CST | Sleep duration | 0.08 | 0.33 | 0.20 | | 0.02 | -0.18 | | 0.03 |  | |  |  |
| ATR | Sleep duration | 0.06 | 0.40 | 0.12 | | 0.16 | -0.10 | | 0.20 |  | |  |  |
| Uncinate | Sleep duration | 0.02 | 0.84 | -0.02 | | 0.83 | -0.09 | | 0.33 |  | |  |  |
| Forceps major | Sleep duration | 0.00 | 0.997 | 0.11 | | 0.19 | -0.09 | | 0.31 |  | |  |  |
| Forceps minor | Sleep duration | 0.12 | 0.12 | 0.14 | | 0.09 | -0.13 | | 0.16 |  | |  |  |
| Corpus callosum | Sleep duration | 0.10 | 0.17 | 0.17 | | 0.05 | -0.17 | | 0.04 |  | |  |  |
| ILF | Sleep duration | -0.02 | 0.78 | 0.21 | | 0.02 | -0.21 | | 0.01 |  | |  |  |
| IFO | Sleep duration | 0.03 | 0.74 | 0.12 | | 0.14 | -0.12 | | 0.12 |  | |  |  |
| SLF | Sleep duration | 0.12 | 0.15 | 0.24 | | 0.01 | -0.18 | | 0.04 |  | |  |  |
| SCS | Sleep duration | 0.10 | 0.21 | 0.11 | | 0.23 | -0.05 | | 0.54 |  | |  |  |
| SIFC | Sleep duration | 0.11 | 0.15 | -0.02 | | 0.82 | -0.11 | | 0.21 |  | |  |  |
| IFSF | Sleep duration | 0.10 | 0.21 | 0.12 | | 0.21 | -0.11 | | 0.22 |  | |  |  |
| Thalamus | Sleep duration | -0.03 | 0.75 | -0.05 | | 0.62 | -0.02 | | 0.79 | 0.05 | | 0.59 |  |
| Caudate | Sleep duration | 0.01 | 0.93 | -0.04 | | 0.61 | -0.03 | | 0.74 | 0.02 | | 0.82 |  |
| Putamen | Sleep duration | 0.07 | 0.50 | 0.15 | | 0.13 | -0.01 | | 0.96 | -0.08 | | 0.42 |  |
| Hippocampus | Sleep duration | -0.10 | 0.23 | -0.01 | | 0.89 | -0.14 | | 0.11 | 0.15 | | 0.08 |  |
| Amygdala | Sleep duration | -0.10 | 0.20 | 0.03 | | 0.72 | -0.19 | | 0.03 | 0.21 | | 0.02 |  |

Beta estimates are for models including only linear sleep duration, as including squared sleep duration did not significantly (*p*<0.05) improve model fit. Models are adjusted for age, sex, education, alcohol consumption, smoking, physical activity, hypertension, BMI, depression, and sleep medication use. HI was not computed in white matter tracts. *P*-values are unadjusted. Linear or squared sleep duration terms did not reach significance after Bonferroni correction for multiple comparisons (*p*<0.017 for all fibers, *p*<0.001 for fiber tracts, *p*<0.003 for subcortical regions). ATR, anterior thalamic radiation; CST, corticospinal tract; HI, hindered isotropic; IF, isotropic free water; IFSF, inferior frontal superior frontal; ILF, inferior longitudinal fasciculus, IFO, inferior frontal occipital; ND, neurite density; RI, restricted isotropic, SCS, superior corticostriatal, SIFC, superior inferior frontal cortex; SLF, superior longitudinal fasciculus

**Table S7**. Standardized beta estimates for fully adjusted regression models assessing associations between sleep duration 9 years before MRI and brain microstructure.

|  |  | **RI** | | **ND** | | **IF** | | **HI** | |
| --- | --- | --- | --- | --- | --- | --- | --- | --- | --- |
| **Region** | **Predictor** | **Beta** | ***p*-value** | **Beta** | ***p*-value** | **Beta** | ***p*-value** | **Beta** | ***p*-value** |
| All fibers | Sleep duration | 0.04 | 0.54 | 0.07 | 0.34 | -0.10 | 0.15 |  |  |
| Fornix | Sleep duration | 0.06 | 0.34 | 0.07 | 0.31 | -0.10 | 0.11 |  |  |
| Cingulum | Sleep duration | -0.03 | 0.73 | 0.13 | 0.14 | -0.09 | 0.29 |  |  |
| Parahippocampal cingulum | Sleep duration | -0.03 | 0.66 | 0.04 | 0.61 | -0.08 | 0.39 |  |  |
| CST | Sleep duration | 0.10 | 0.19 | 0.04 | 0.61 | -0.11 | 0.15 |  |  |
| ATR | Sleep duration | 0.05 | 0.47 | 0.05 | 0.51 | -0.14 | 0.04 |  |  |
| Uncinate | Sleep duration | 0.03 | 0.64 | 0.03 | 0.68 | -0.13 | 0.10 |  |  |
| Forceps major | Sleep duration | -0.07 | 0.38 | 0.08 | 0.31 | -0.09 | 0.25 |  |  |
| Forceps minor | Sleep duration | 0.04 | 0.57 | 0.12 | 0.11 | -0.09 | 0.23 |  |  |
| Corpus callosum | Sleep duration | 0.03 | 0.70 | 0.08 | 0.25 | -0.08 | 0.27 |  |  |
| ILF | Sleep duration | -0.04 | 0.59 | 0.07 | 0.40 | -0.14 | 0.05 |  |  |
| IFO | Sleep duration | 0.00 | 0.96 | 0.07 | 0.34 | -0.11 | 0.10 |  |  |
| SLF | Sleep duration | 0.05 | 0.51 | 0.08 | 0.37 | -0.04 | 0.59 |  |  |
| SCS | Sleep duration | 0.08 | 0.30 | -0.01 | 0.88 | -0.02 | 0.78 |  |  |
| SIFC | Sleep duration | 0.11 | 0.09 | 0.01 | 0.91 | -0.12 | 0.15 |  |  |
| IFSF | Sleep duration | 0.07 | 0.34 | -0.02 | 0.78 | 0.00 | 0.99 |  |  |
| Thalamus | Sleep duration | -0.06 | 0.47 | -0.07 | 0.42 | -0.09 | 0.26 | 0.11 | 0.22 |
| Caudate | Sleep duration | -0.02 | 0.82 | -0.05 | 0.48 | -0.12 | 0.14 | 0.13 | 0.09 |
| Putamen | Sleep duration | 0.01 | 0.96 | 0.08 | 0.40 | -0.10 | 0.27 | 0.02 | 0.83 |
| Hippocampus | Sleep duration | -0.08 | 0.34 | 0.06 | 0.47 | -0.14 | 0.06 | 0.16 | 0.05 |
| Amygdala | Sleep duration | -0.09 | 0.25 | 0.02 | 0.80 | -0.14 | 0.09 | 0.16 | 0.06 |

Beta estimates are for models including only linear sleep duration, as including squared sleep duration did not significantly (*p*<0.05) improve model fit. Models are adjusted for age, sex, education, alcohol consumption, smoking, physical activity, hypertension, BMI, depression, and sleep medication use. HI was not computed in white matter tracts. *P*-values are unadjusted. Linear or squared sleep duration terms did not reach significance after Bonferroni correction for multiple comparisons (*p*<0.017 for all fibers, *p*<0.001 for fiber tracts, *p*<0.003 for subcortical regions). ATR, anterior thalamic radiation; CST, corticospinal tract; HI, hindered isotropic; IF, isotropic free water; IFSF, inferior frontal superior frontal; ILF, inferior longitudinal fasciculus, IFO, inferior frontal occipital; ND, neurite density; RI, restricted isotropic, SCS, superior corticostriatal, SIFC, superior inferior frontal cortex; SLF, superior longitudinal fasciculus

**Table S8**. Participant characteristics (mean±SD unless otherwise noted) at MRI by sex.

| **Variable** | **Women (*N*=71)** | **Men (*N*=46)** | **Sex difference** |
| --- | --- | --- | --- |
| Age at MRI (years) | 76.2±8.0 | 77.1±8.1 | *F*(1,115)=0.35, *p*=0.55 |
| PSQI | 5.5±3.3 | 4.9±3.0 | *F*(1,115)=1.10, *p*=0.30 |
| Hours asleep (25 years before MRI) | 7.2±0.8 | 7.1±0.8 | *F*(1,99)=0.48, *p*=0.49 |
| Hours asleep (15 years before MRI) | 7.4±0.9 | 7.4±0.8 | *F*(1,97)=0.05, *p*=0.82 |
| Hours asleep (9 years before MRI) | 7.4±0.8 | 7.2±1.0 | *F*(1,112)=1.34, *p*=0.25 |
| Education (years) | 14.4±2.0 | 15.8±2.0 | ***F*(1,115)=13.10, *p*<0.001** |
| Sleep medication use (at any sleep assessment) | *N*=27 (24%) | *N*=9 (20%) | *x^2^*(1)=0.29, *p*=0.59 |
| Hypertension | *N*=39 (55%) | *N*=31 (67%) | *x^2^*(1)=1.80, *p*=0.18 |
| *APOE* (ε4-carrier) | *N*=19 (29%) | *N*=8 (17%) | *x^2^*(1)=1.92, *p*=0.17 |
| Exercise (3+ times/week) | *N*=54 (77%) | *N*=36 (78%) | *x^2^*(1)=0.02, *p*=0.89 |
| Body mass index (BMI, kg/m^2^) | 24.8±3.9 | 27.1±3.1 | ***F*(1,115)=10.63, *p*=0.001** |
| Smoking (ever) | *N*=28 (40%) | *N*=23 (50%) | *x^2^*(1)=1.13, *p*=0.29 |
| Alcohol (current drinker) | *N*=63 (90%) | *N*=40 (87%) | *x^2^*(1)=0.26, *p*=0.62 |
| Beck depression inventory | 4.1±3.9 | 3.9±3.1 | *F*(1,115)=0.10, *p*=0.75 |
| Marital status (married) | *N*=47 (67%) | *N*=43 (93%) | ***x^2^*(1)=11.07, *p*<0.001** |

**BOLD** = *p*<0.05

**Table S9**. Standardized beta estimates for fully adjusted regressions assessing associations between sleep quality (PSQI) or duration and white matter or subcortical volume (adjusted for intracranial volume).

|  | **PSQI** | | **Sleep duration**  **25 years before MRI** | | | **Sleep duration**  **15 years before MRI** | | **Sleep duration**  **9 years before MRI** | |
| --- | --- | --- | --- | --- | --- | --- | --- | --- | --- |
| **Region** | **Beta** | ***p*-value** | | **Beta** | ***p*-value** | **Beta** | ***p*-value** | **Beta** | ***p*-value** |
| White matter | -0.05 | 0.59 | | -0.05 | 0.55 | -0.02 | 0.82 | 0.08 | 0.33 |
| Thalamus | -0.18 | 0.10 | | 0.03 | 0.73 | 0.03 | 0.79 | 0.09 | 0.32 |
| Caudate | 0.21 | 0.06 | | -0.07 | 0.48 | 0.02 | 0.88 | 0.05 | 0.59 |
| Putamen | -0.14 | 0.18 | | 0.04 | 0.67 | 0.03 | 0.74 | -0.11 | 0.26 |
| Hippocampus | -0.09 | 0.35 | | 0.04 | 0.62 | -0.03 | 0.78 | 0.08 | 0.31 |
| Amygdala | 0.04 | 0.68 | | -0.08 | 0.42 | -0.09 | 0.34 | -0.02 | 0.82 |

Beta estimates are for models including only linear PSQI or sleep duration terms, as including squared sleep terms did not significantly (*p*<0.05) improve model fit. Models are adjusted for age, sex, education, alcohol consumption, smoking, physical activity, hypertension, BMI, and depression. Models for sleep duration are additionally adjusted for sleep medication use. *P*-values are unadjusted. PSQI, Pittsburg sleep quality index
